# DroneRF dataset: A dataset of drones for RF-based detection, classification and identification

**DOI:** 10.1016/j.dib.2019.104313

**Published:** 2019-08-26

**Authors:** MHD Saria Allahham, Mohammad F. Al-Sa'd, Abdulla Al-Ali, Amr Mohamed, Tamer Khattab, Aiman Erbad

**Affiliations:** a Qatar University, Department of Computer Science and Engineering, Doha, Qatar; b Laboratory of Signal Processing, Tampere University of Technology, Tampere, Finland; c Qatar University, Department of Electrical Engineering, Doha, Qatar

**Keywords:** UAV detection, Drone identification, Classification, Anti-drone systems

## Abstract

Modern technology has pushed us into the information age, making it easier to generate and record vast quantities of new data. Datasets can help in analyzing the situation to give a better understanding, and more importantly, decision making. Consequently, datasets, and uses to which they can be put, have become increasingly valuable commodities. This article describes the DroneRF dataset: a radio frequency (RF) based dataset of drones functioning in different modes, including off, on and connected, hovering, flying, and video recording. The dataset contains recordings of RF activities, composed of 227 recorded segments collected from 3 different drones, as well as recordings of background RF activities with no drones. The data has been collected by RF receivers that intercepts the drone's communications with the flight control module. The receivers are connected to two laptops, via PCIe cables, that runs a program responsible for fetching, processing and storing the sensed RF data in a database. An example of how this dataset can be interpreted and used can be found in the related research article “RF-based drone detection and identification using deep learning approaches: an initiative towards a large open source drone database” (Al-Sa'd et al., 2019).

**Table:** Specifications Table

Subject area	*Engineering.*
More specific subject area	*RF communications and Data collection.*
Type of data	*Tables and Figures.*
How data was acquired	*Using two RF Receivers (NI-USRP2943R) in a laboratory setting.*
Data format	*Raw.*
Experimental factors	*To capture the whole 2.4GHz bandwidth, we have used 2 RF receivers. Each RF receiver has a maximum instantaneous bandwidth of 40 MHz, so both receivers must be operating simultaneously to at least capture a technology spectrum such as WiFi (i.e. 80 MHz) where the first receiver captures the lower half of the frequency band, and the second, records the upper half.*
Experimental features	*We systematically collect, analyze, and record raw RF signals of different drones under different flight modes such as: off, on and connected, hovering, flying, and video recording.*
Data source location	*Department of Computer Science and Engineering, College of Engineering, Qatar University, Doha, Qatar.*
Data accessibility	Al-Sa'd, Mohammad; Allahham, Mhd Saria; Mohamed, Amr; Al-Ali, Abdulla; Khattab, Tamer; Erbad, Aiman (2019), “DroneRF dataset: A dataset of drones for RF-based detection, classification, and identification”, Mendeley Datasets, v1 https://doi.org/10.17632/f4c2b4n755.1[8]
Related research article	Mohammad F. Al-Sa'd, Abdulla Al-Ali, Amr Mohamed, Tamer Khattab, and Aiman Erbad, “RF-based drone detection and identification using deep learning approaches: an initiative towards a large open source drone database”, *Future Generation Computer Systems*, 2019.

**Table:** 

**Value of the data** • The droneRF dataset can be used to develop new techniques for drones' detection and identification, or as a critical building block in a large-scale anti-drone system that includes other functions such as drones' intrusion detection, tracking, jamming, and activity logging. • DroneRF helps in understanding the signatures of different drones operating in different modes (see section 1.6 for details about the drones' flight modes) based on their radio frequency signal characteristics. • DroneRF can inspire new methods for detecting the drones' existence, and possibly identifying the drones' make, type, etc.

## 1 Data

In this article, we present an RF based dataset of drones functioning in different modes. The dataset consists of recorded segments of RF background activities with no drones, and segments of drones operating in different modes such as: off, on and connected, hovering, flying, and video recording (see Fig. 4, Fig. 5, Fig. 6). The records are 10.25 seconds of RF background activities and approximately 5.25 seconds of RF drone communications for each flight mode. This has produced a drone RF database with over 40 GB of data encompassing various RF signatures. There are in total 227 segments (see Table 1), each segment consists of two equally sized parts with each part containing 1 million samples, making in total 454 record files. The samples in the segments represent the amplitude of the acquired row RF signals in the time domain. The segments in the database [2] are stored as comma-separated values (csv) files, this makes the drone RF database easy to load and interpret on any preferred software. Metadata for each segment in the database is included within its filename. It contains the segment Binary Unique Identifier (BUI) to differentiate between the drone's mode (see Fig. 7), followed by a character to determine if it is the first or second half of the segment, and its segment number. For instance, the third segment of the second half of the RF spectrum with BUI = 11010, Phantom drone with flight mode number 3, will have the following filename: “11010H3.csv”.

**Fig. 1:**
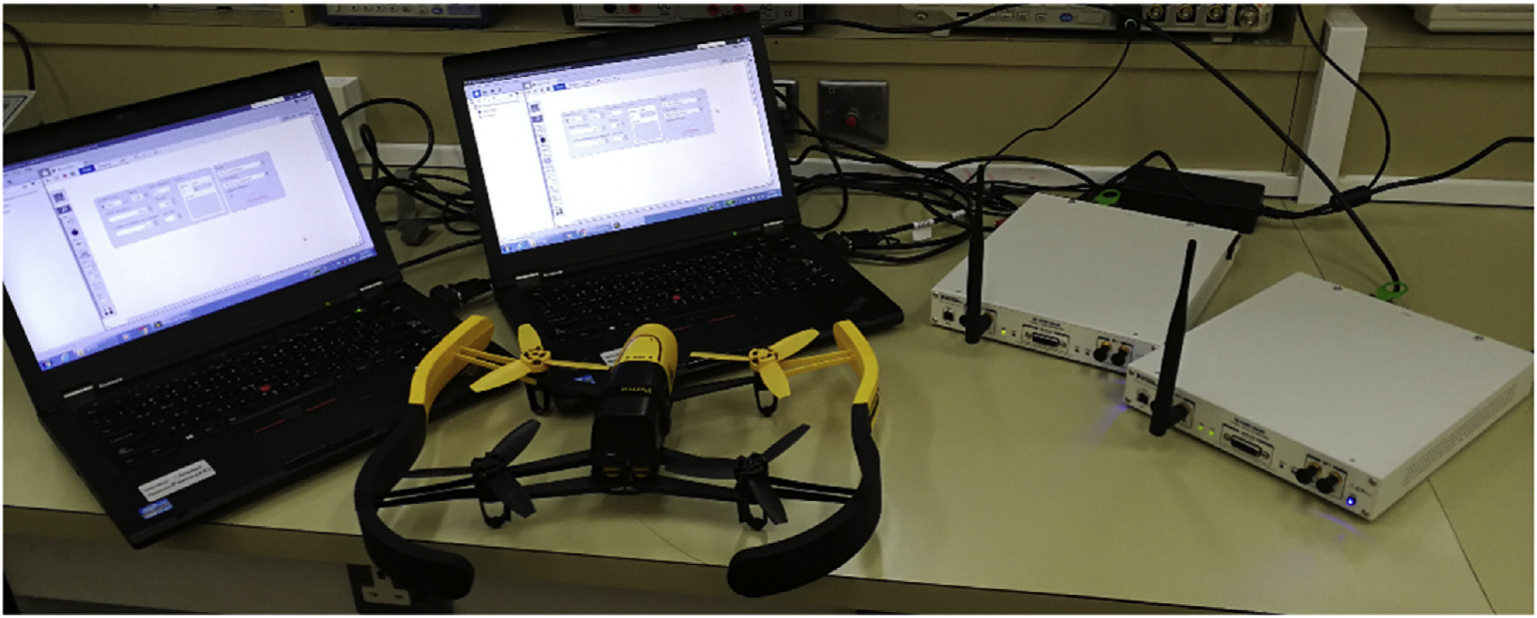
Experimental setup for the RF database development. The Bebop drone is shown on the middle, the NI-USRP RF receivers are shown on the right and are connected to the laptops, shown on the left, via the PCIe connectors.

**Fig. 2:**
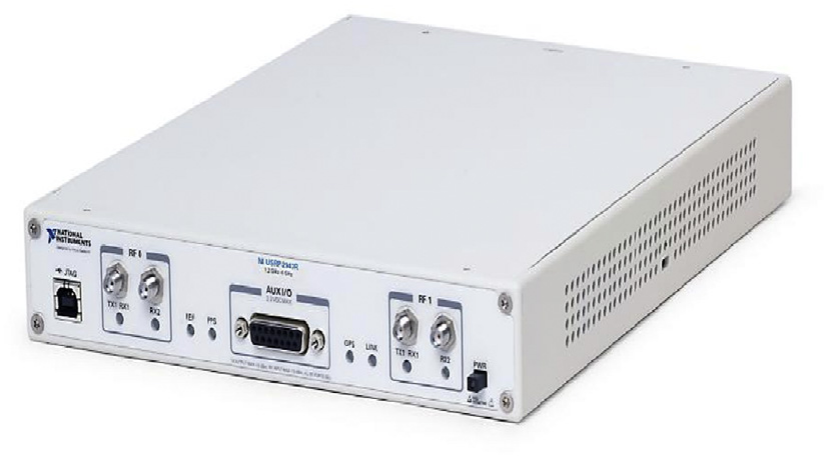
NI USRP-2943R RF receiver [4].

**Fig. 3:**
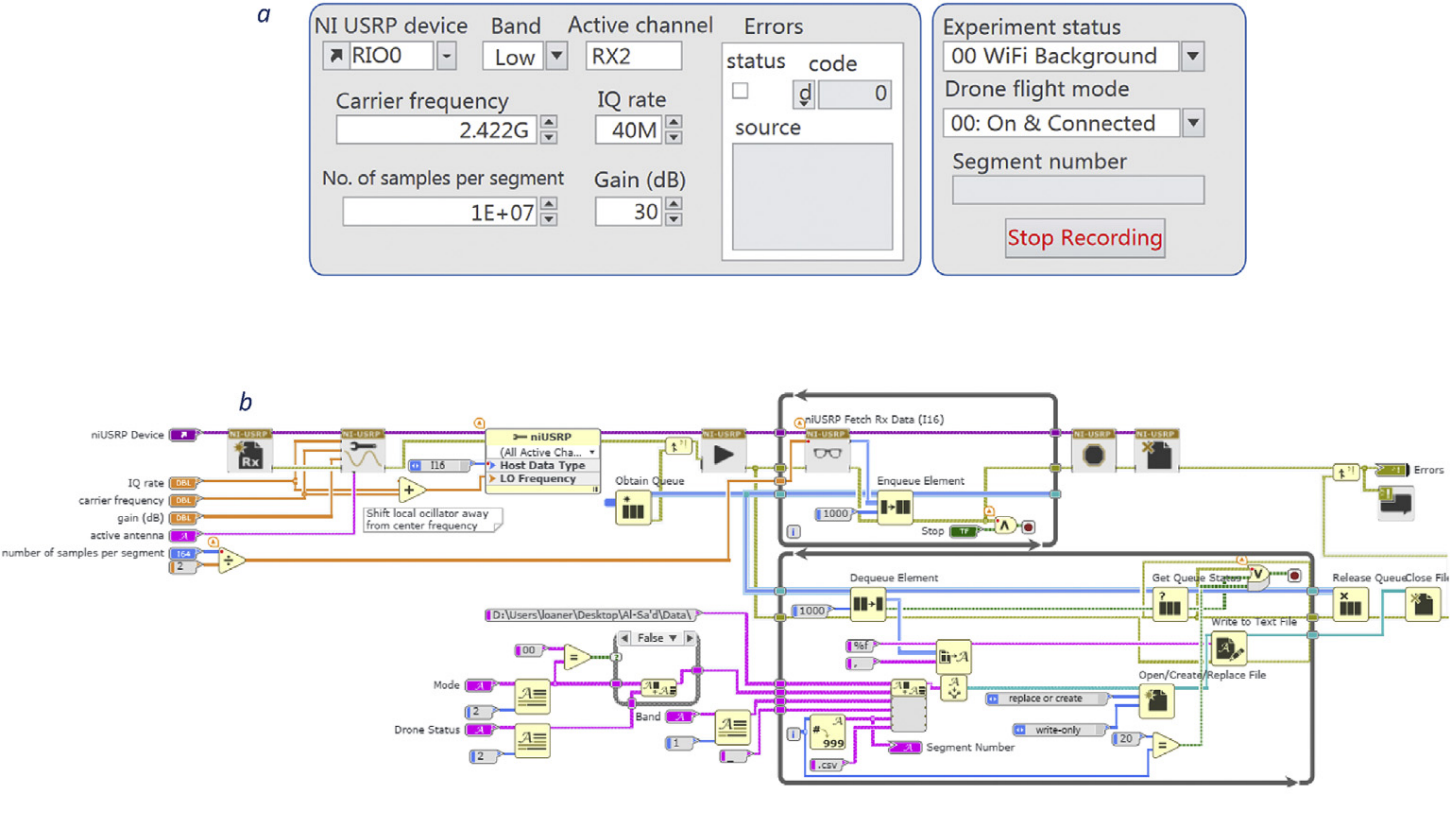
a: Front panel of the LabVIEW program installed on the laptops to capture the drones' RF communication [1]. b: Block diagram of LabVIEW program [1].

**Fig. 4:**
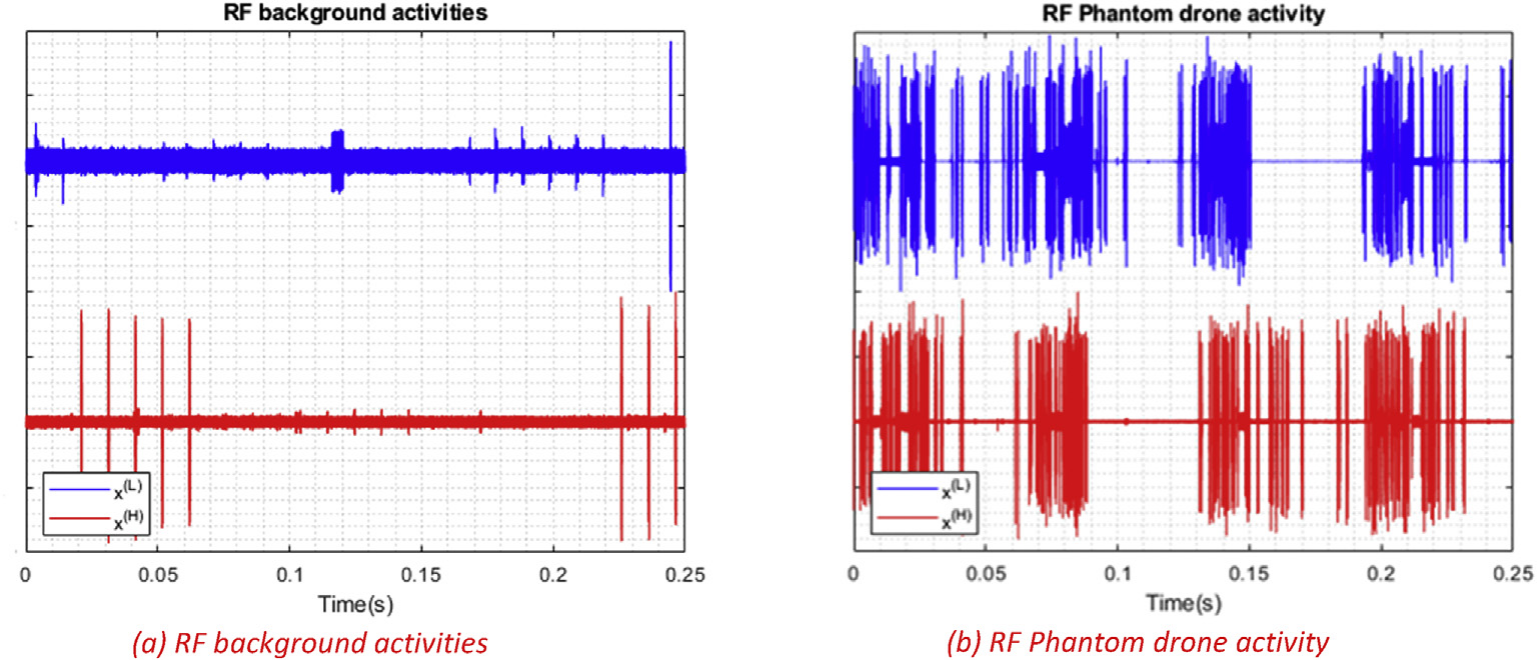
RF activities plots with normalized amplitudes between −1 and 1. (a) shows segment number 13 of the acquired RF background activities, (b) shows segment number 10 of the acquired Phantom drone activity.

**Fig. 5:**
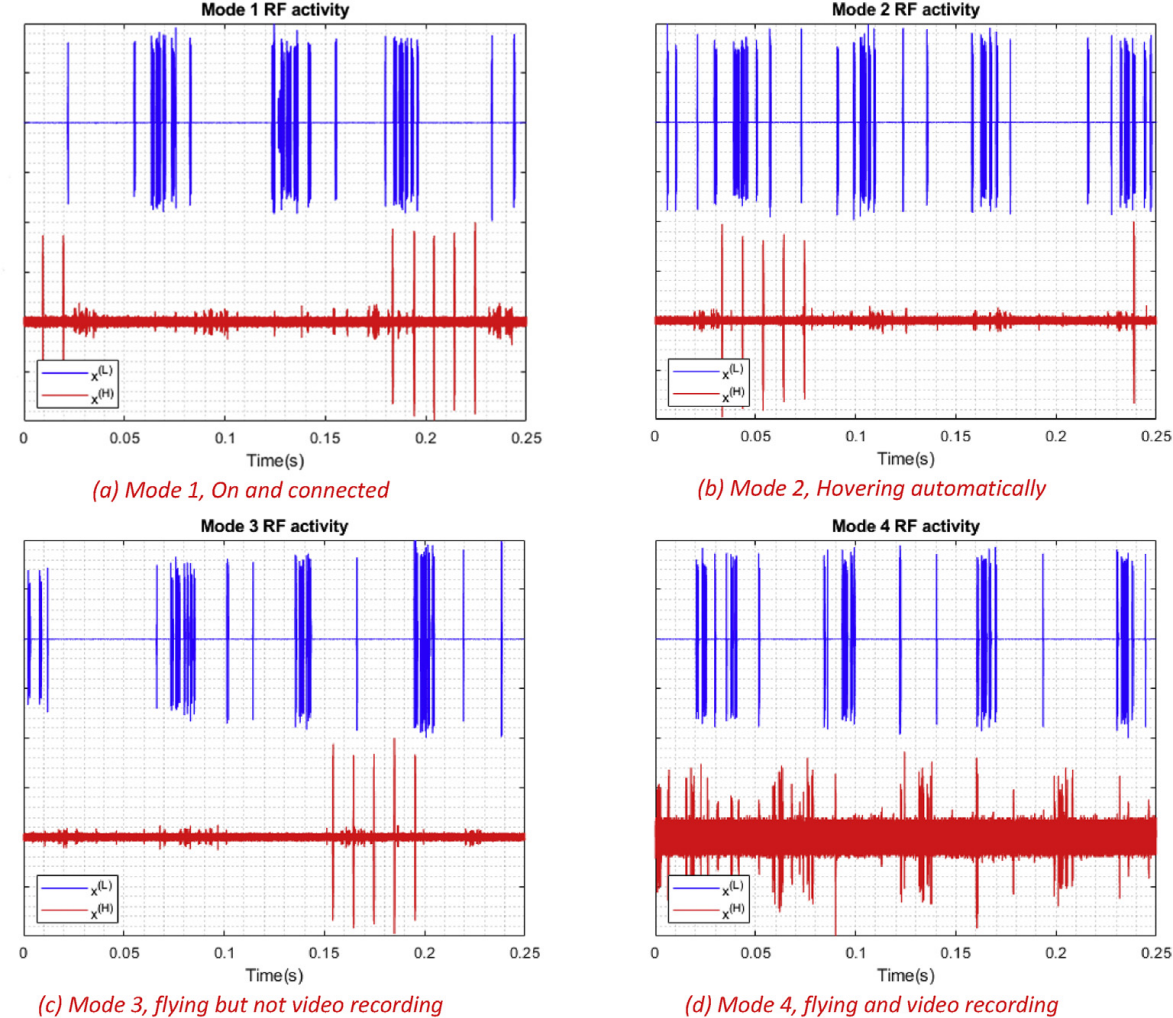
Different snippets of RF activities for different flight modes for the Bebop drone with normalized amplitude between 1 and -1. Each figure shows the segment number 1 of each flight mode.

**Fig. 6:**
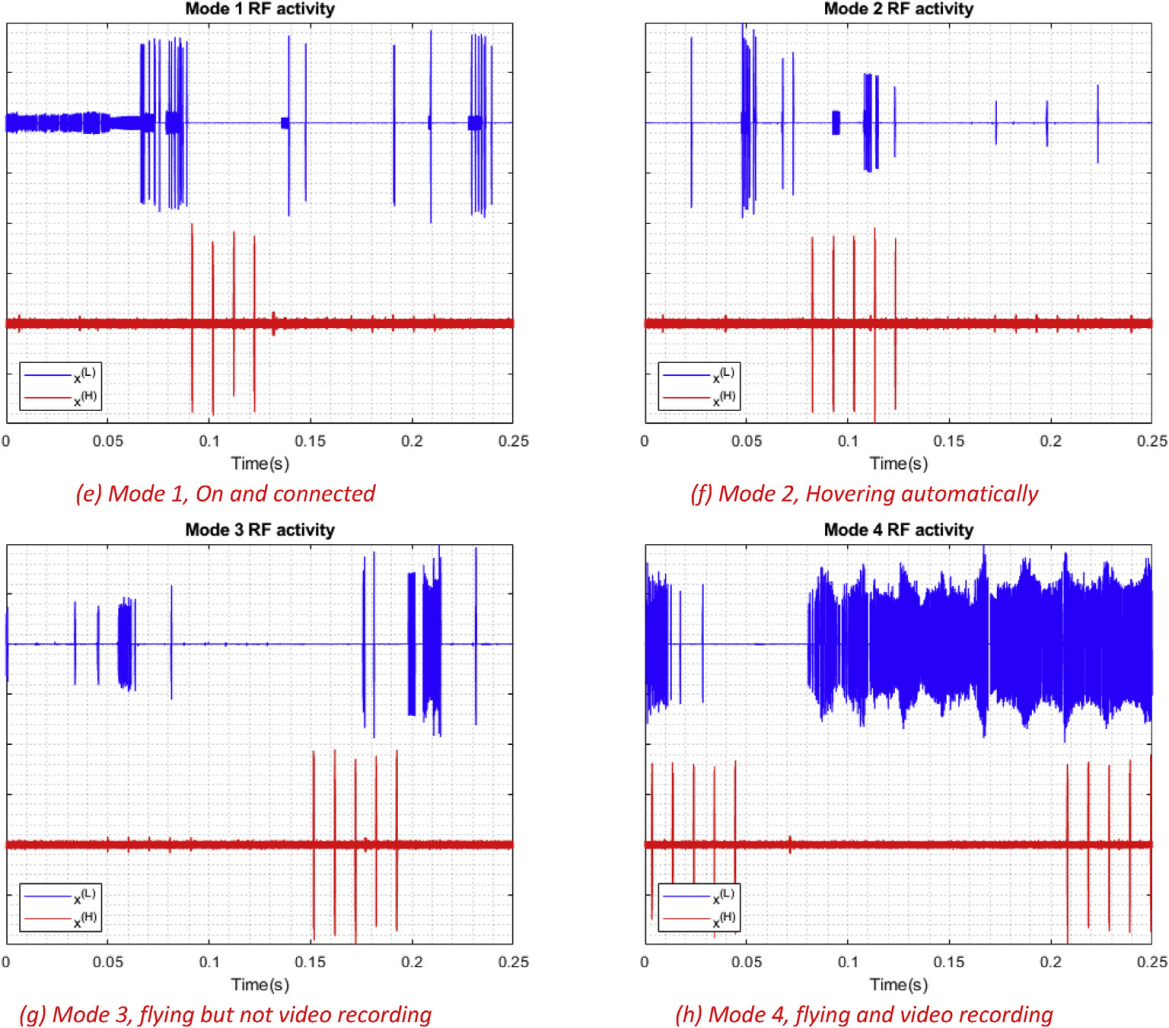
Different snippets of RF activities for different flight modes for the AR drone with normalized amplitude between 1 and -1. Each figure shows the segment number 1 of each flight mode.

**Table 1:** Details of the developed drone RF database showing the number of raw samples and segments for each drone type.

Drone Type	Segments	Samples	Ratio
Bepop	84	$1,680\times 10^{6}$	37.00%
AR	81	$1,620\times 10^{6}$	35.68%
Phantom	21	$420\times 10^{6}$	9.25%
No Drone	41	$820\times 10^{6}$	18.06%

**Fig. 7:**
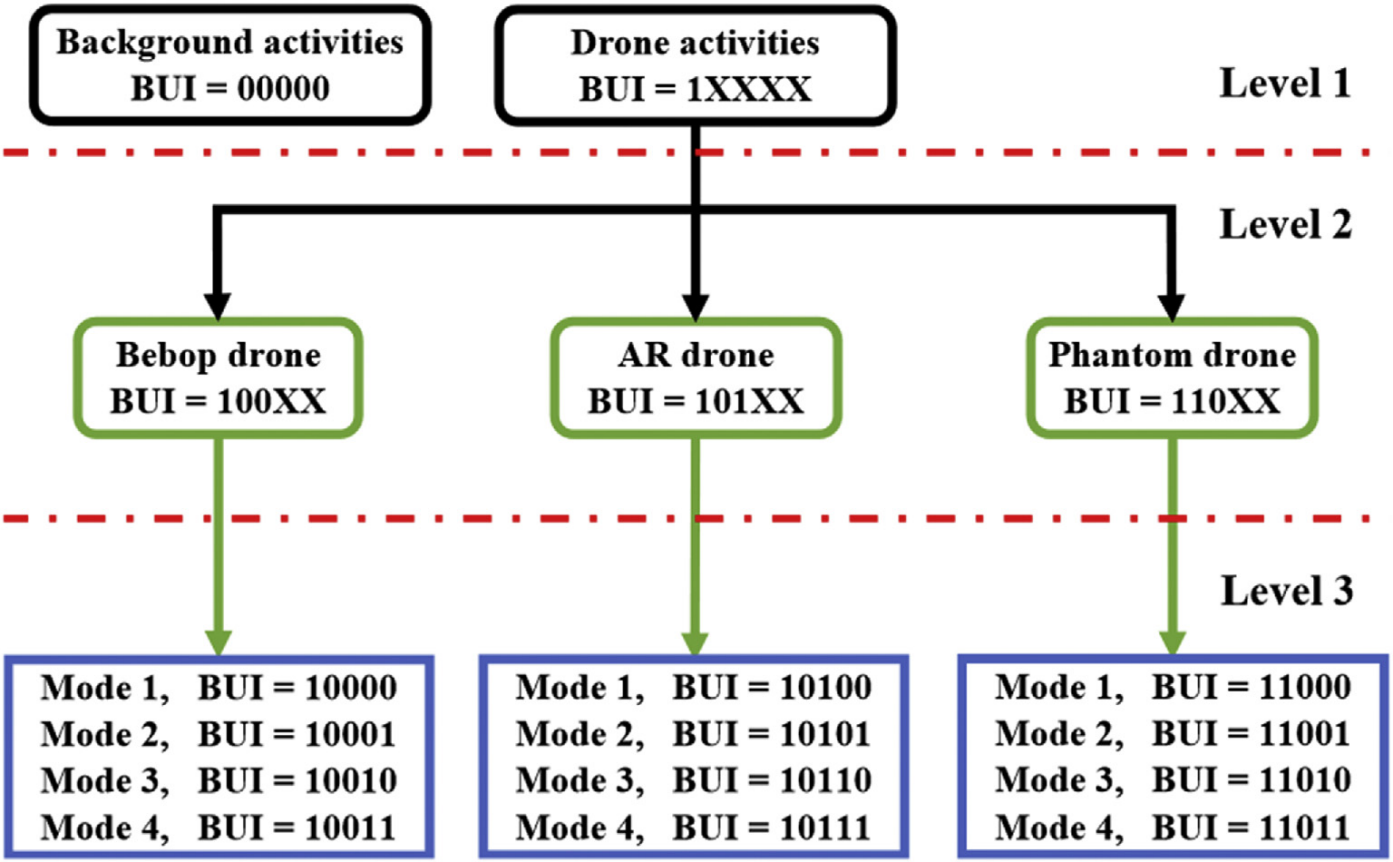
Experiments to record drones RF signatures organized in a tree manner consisting of three levels. The horizontal dashed red lines define the levels. BUI is a Binary Unique Identifier for each component to be used in labelling [1].

## 2 Experimental design, materials, and methods

### 2.1 Equipment

In our experiment, flight controllers and mobile phones were used to send and receive the RF commands, to and from the drones under analysis to alter their flight mode. Controlling the drones by a mobile phone requires mobile applications that are specifically developed for each drone. “FreeFlight Pro”, “AR.FreeFlight”, and “DJI Go” are free mobile applications developed to control the Bebop, AR, and Phantom drones, respectively. RF commands intercepted by two RF receivers, described in the next section, each receiver communicates with a laptop (CPU Intel Core i5-520M 2.4 GHz, 4 GB RAM, running Windows 7 64-bit) through PCIe cable (see Fig. 1), doing data fetching, processing and storing are performed by programs we designed in LabVIEW Communications System Design Suite [3].

### 2.2 Sensing and capturing RF signals

RF sensing and capturing process required RF receivers, to intercept the drone communication with its flight controller, connected to laptops that are responsible for fetching, processing and storing the recorded RF signals in the database. The drones that have been used in the experiment operates differently in terms of connectivity, (e.g. Bebop and Phantom use WiFi 2.4 GHz and 5 GHz with different bandwidths). For more details, one can read the full specification in [5], [6], [7]. In this work, it is assumed that all drones use WiFi operated at 2.4 GHz. Hence, there are some minimal assumptions. However, the drone operating frequency can be detected using various methods such as passive frequency scanning.

First, raw RF samples are acquired using two National Instruments USRP-2943 (NI-USRP) software defined radio reconfigurable devices, shown in Fig. 2: NI USRP-2943R RF receiver. Table 2 lists the NI-USRP RF receivers’ specifications. Since each RF receiver has a maximum instantaneous bandwidth of 40 MHz, both receivers must be operated simultaneously to at least capture a technology spectrum such as WiFi (i.e. 80 MHz) where the first receiver captures the lower half of the frequency band, and the second, records the upper half (see Fig. 4). After that, captured RF data is transferred from the NI-USRP receivers to two standard laptops via Peripheral Component Interconnect Express (PCIe) interface kits. Finally, data fetching, processing and storing are performed by programs we designed in LabVIEW Communications System Design Suite. The programs are designed in a standard LabVIEW manner using front panel and block diagram environments. Fig. 3b shows the block diagram that demonstrates all the functions and operations used to fetch process and store the RF data.

**Table 2:** Specifications of the USRP-2943 40 MHz RF receivers.

Number of channels	2
Frequency range	1.2 GHz–6 GHz
Frequency step	<1 KHz
Gain range	0 dB–37.5 dB
Maximum instantaneous bandwidth	40 MHz
Maximum I/Q sample rate	200 MS/s
ADC resolution	14 bits

As demonstrated in Fig. 3a, by using the front panel, one can alter the captured band; lower half or upper half of the RF spectrum, carrier frequency, IQ rate, number of samples per segment, gain, and activate a specific channel of the NI-USRP receiver. In addition, one can select different flight modes and experiments to build a comprehensive database.

### 2.3 Experimental setup

The setup is shown in Fig. 1. To conduct any experiment using this setup, one must perform the following tasks carefully and sequentially. If you are recording RF background activities, perform tasks 4–7:

1. Turn on the drone under analysis and connect to it using a mobile phone or a flight controller.
2. In case the utility of a mobile phone as a controller, start the mobile application to control the drone and to change its flight mode.
3. Check the drone connectivity and operation by performing simple takeoff, hovering, and landing tests.
4. Turn on the RF receivers to intercept all RF activities and to transfer those to the laptops via the PCIe connectors.
5. Open the LabVIEW programs, installed on the laptops, and select appropriate parameters depending on your experiment and requirements.
6. Start the LabVIEW programs to fetch, process and store RF data segments.
7. Stop the LabVIEW programs when you are done with the experiment.
8. For a different flight mode, go back to step 6, and for different drones go back to step 1.

### 2.4 Experiments

The RF drone database is populated with the required signatures by conducting experiments organized in a tree manner with three levels as demonstrated in Fig. 7. The first level consists of the following branches to train and assess the drone detection system:

• Drones are off; RF background activities are recorded (see Fig. 4a).
• Drones are on; drones RF activities are recorded (see Fig. 4b).

The second level includes experiments that are conducted on the three drones under analysis: Bebop, AR, and the Phantom drones, to train and assess the drone identification system. Finally, the third level expands its predecessor by explicitly controlling the flight mode of each drone under analysis to assess the identification system ability in determining the flight mode of intruding drones.

• On and connected to the controller (Mode 1, see Fig. 5a).
• Hovering automatically with no physical intervention nor control commands from the controller. Hovering altitude is determined by the drone manufacturer (approximately 1 m) (Mode 2, see Fig. 5b).
• Flying without video recording. Note that the drone must not hit any obstacles in this experiment to avoid warning signals (Mode 3, see Fig. 5c).
• Flying with video recording (Mode 4, see Fig. 5d).

Note that the drone must not hit any obstacles in this experiment to avoid warning signals. The former experiments are conducted by following the steps summarized in the previous section.

### 2.5 RF database labeling

The BUI is used to label the RF database entries according to the conducted experiment, drone type, and its specific flight mode, see Fig. 7: Experiments to record drones RF signatures organized in a tree manner consisting of three levels. The horizontal dashed red lines define the levels. BUI is a Binary Unique Identifier for each component to be used in labelling. The BUI is comprised of two binary numbers concatenated such that: BUI = [msBUI, lsBUI], msBUI is the most significant part of the BUI representing the experiment and drone type, levels one and two, while lsBUI is the least significant part of the BUI representing the drone flight mode, third level. The BUI length L is determined using the total number of experiments E, the total number of drones D, and the total number of flight modes F, as follows:

$$L=\;\log_{2}\left(E\right)+\log_{2}\left(D\right)+\log_{2}\left(F\right)$$

where in this work, E=2,D=3 and F=4; therefore, L=5. Extending the developed database using other experiments, drones, or flight modes can be easily done by increasing E,Dor F, respectively.

### 2.6 Visualizing the data

To visualize the data, various software can be used. As mentioned before, each flight mode recording is composed of segments, each segment is split into two parts. In order to plot a segment, the two parts should be loaded into the software work-space together (E.g. 11000L_3, 11000H_3). After loading the data, amplitude normalization can be done for a better visualization, and that's by dividing all the samples in the segment by its maximum absolute value to end up with values between 1 and -1. If needed for data analysis, frequency samples can also be calculated using Discrete Fourier transform (DFT) of each recorded segment coming from both receivers [1]. Fig. 4, Fig. 5, Fig. 6 were generated by shifting the samples of one part e.g. 11000L_3 up by adding 1, while shifting the other part down by subtracting 1.
